# Design of Low-Loss Acoustic Delay Lines Enabled by Dual-Mode Interface Acoustic Waves in SiO_2_/ZnO/IDT/SU-8/SiO_2_ Structures

**DOI:** 10.3390/mi17070781

**Published:** 2026-06-27

**Authors:** Cinzia Caliendo, Farouk Laidoudi, Fabio Lo Castro

**Affiliations:** 1 Institute for Photonics and Nanotechnology, National Research Council of Italy, IFN-CNR, Via del Fosso del Cavaliere 100, 00133 Rome, Italy; 2 Research Center in Industrial Technologies (CRTI), P.O. Box 64, Cheraga, Algiers 16014, Algeria; f.laidoudi@crti.dz; 3 Institute of Marine Engineering, National Research Council of Italy, CNR-INM—Section of Acoustics and Sensors “O.M. Corbino”, Via del Fosso del Cavaliere 100, 00133 Rome, Italy; fabio.locastro@cnr.it

**Keywords:** Interface Acoustic Wave (IAW), downward IAW, upward IAW, ZnO, SiO_2_, SU-8

## Abstract

The present work explores the modelling and design of Interface Acoustic Wave (IAW)-based delay lines in SiO_2_/ZnO (4 µm)/SU-8/SiO_2_ multilayer stacks and demonstrates that, by properly tailoring the acoustic wavelength and the SU-8 layer thickness, IAW delay lines can achieve performances comparable to, and in some cases superior to, those of conventional Surface Acoustic Wave (SAW) delay lines based on SiO_2_/ZnO (4 µm) structures. In particular, the proposed devices exhibited untuned insertion losses down to 12 dB, propagation losses as low as 0.052 dB/λ, and electromechanical coupling coefficients *K*^2^ approaching 4%, exceeding those calculated for the corresponding SAW devices. The obtained results support the feasibility of compact, high-performance, and potentially packageless acoustic-wave devices for future telecommunications and sensing applications, especially in harsh or contamination-prone environments.

## 1. Introduction

Electroacoustic devices based on surface acoustic waves (SAWs) have been extensively investigated for sensing, signal-processing, and microelectronic applications because of their compactness, high sensitivity, and compatibility with planar fabrication technologies [1,2]. However, the strong confinement of acoustic energy near the free surface, while responsible for their high sensitivity, also makes SAW devices intrinsically vulnerable to environmental perturbations such as humidity, contaminants, temperature and pressure fluctuations. Consequently, conventional SAW devices generally require protective packaging, which increases device complexity and fabrication cost. In this context, interface acoustic waves (IAWs), which propagate along the boundary between two bonded bulk solids—rather than at a free surface—can represent an attractive alternative. Because the acoustic energy is guided at buried interfaces, IAW devices can potentially mitigate surface-contamination effects while also exhibiting phase velocities higher than those of conventional SAWs. The concept of acoustic boundary waves was first proposed by T. Irino and collaborators in 1988 [3], who demonstrated interface-guided wave propagation in SiO_2_/ZnO/SiO_2_ multilayers with propagation losses comparable to those of Rayleigh waves. Renewed interest has recently been devoted to IAWs supported by bonded multilayer structures employing an intermediate SU-8 adhesive layer, such as LiNbO_3_/SU-8/Si and, more recently, SiO_2_/ZnO/SU-8/SiO_2_ configurations [4,5,6]. In these systems, interface modes can be interpreted as the evolution of the SAWs supported by the bare substrate toward waves localized at buried interfaces and propagating at higher phase velocities. Previous theoretical and experimental investigations [6] demonstrated that the SiO_2_/ZnO/SU-8/SiO_2_ multilayer supports two distinct IAW families: a downward-confined IAW mainly localized near the lower SiO_2_/ZnO interface and an upward-confined IAW preferentially localized toward the ZnO/SU-8/SiO_2_ upper region. These modes can therefore be regarded as the natural evolution of the conventional Rayleigh wave supported by the reference SiO_2_/ZnO structure once the SU-8/SiO_2_ overlayer is introduced. The existence of these IAWs in SiO_2_/ZnO/SU-8/SiO_2_ structures has already been demonstrated theoretically and experimentally for wavelengths of several tens of micrometers and relatively thick SU-8 bonding layers. However, the corresponding IAW-based delay lines exhibited propagation losses significantly higher than those of conventional SAW delay lines based on SiO_2_/ZnO structures [7]. A systematic investigation of the geometrical parameters governing electromechanical coupling and propagation loss in ZnO-based IAW devices is therefore essential to optimize these multilayer structures for sensing, signal-processing, and telecommunications applications.

One possible sensing application of IAW-based devices is ultra-violet (UV) detection, exploiting both the optical transparency of fused silica at a wavelength of 365 nm and the UV sensitivity of the ZnO piezoelectric layer. Indeed, SAW-based UV sensors have already been successfully demonstrated using ZnO/fused-silica structures, owing to the wide-bandgap and photoconductive properties of ZnO [8,9]. IAW UV sensors could therefore represent a complementary class of acoustic-wave sensors, particularly for applications requiring a protected acoustic propagation path, reduced susceptibility to unwanted surface perturbations, and enhanced robustness in harsh environments.

It has been demonstrated that IAW-based devices can also be employed in telecommunications systems. Reference [10] reviews miniature, high-performance RF filters based on IAWs propagating in rotated YX-LiNbO_3_ structures, reporting minimum insertion losses comparable to those of conventional SAW filters, together with a significantly reduced packaged-device size. The IAW filters developed to date have largely exploited the high electromechanical coupling coefficient of LiNbO_3_, which enables efficient acoustic-wave excitation and low-loss filtering. In contrast, the present work investigates a ZnO-based IAW delay line and indicates that, through appropriate geometrical optimization, these multilayer structures can achieve propagation losses comparable to those of conventional SiO_2_/ZnO SAW devices. Extending the proposed ZnO-based platform from delay lines to IAW resonators and filters, therefore, represents an important future development and will be addressed in a dedicated forthcoming work.

The present study investigates design strategies aimed at improving the performance of IAW-based delay lines implemented in SiO_2_/ZnO/SU-8/SiO_2_ multilayer structures. In particular, the effects of the ZnO and SU-8 thickness-to-wavelength ratios on the electromechanical coupling coefficient, *K*^2^, and wave-propagation loss are analyzed through eigenfrequency and frequency-domain simulations. The results show that reducing the acoustic wavelength to a few micrometers significantly enhances *K*^2^ while substantially decreasing both insertion and propagation losses. Under the optimized conditions considered in this study, propagation losses as low as 0.052 dB/λ were predicted, approaching those of conventional SAW delay lines. At the same time, *K*^2^ values approaching 4% were obtained, exceeding those supported by the reference SiO_2_/ZnO SAW structure having the same wavelength and ZnO-layer thickness. These results support the feasibility of compact, high-performance, and potentially packageless acoustic-wave platforms for future telecommunications and sensing applications in harsh or contamination-prone environments.

## 2. Materials and Methods

A two-dimensional finite-element analysis was carried out to investigate IAW propagation in SiO_2_/ZnO/SU-8/SiO_2_ multilayer structures with fixed ZnO thickness (4 μm), while varying both the acoustic wavelength λ and the SU-8 layer thickness. Eigenfrequency and Frequency Domain studies were performed using the software COMSOL Multiphysics® v. 6.3 (COMSOL AB, Stockholm, Sweden), employing the Solid Mechanics and Electrostatics interfaces under the Piezoelectric Devices multiphysics coupling.

**Figure 1:**
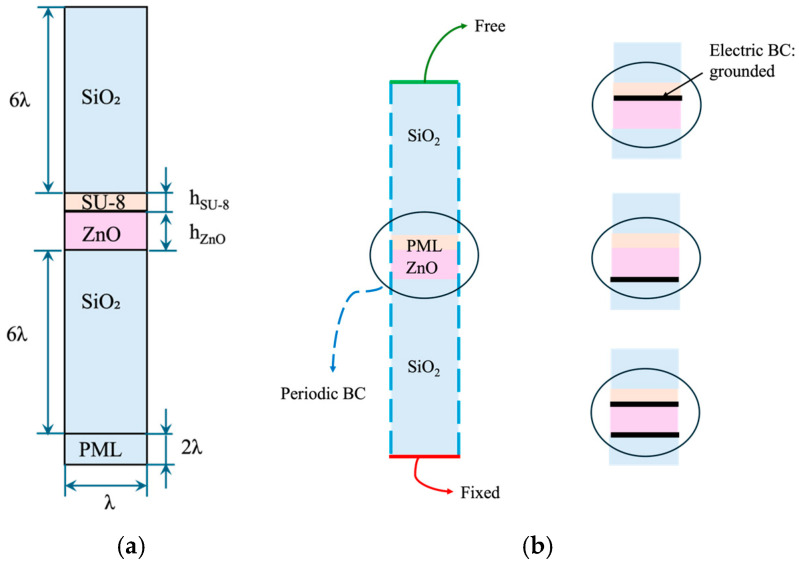
(**a**) Unit cell employed for the eigenfrequency analyses; (**b**) Boundary conditions (BCs) applied to the unit cell. The circular insets illustrate the different electrical boundary conditions adopted for the eigenfrequency calculations. The drawings are not to scale.

Eigenfrequency analyses were conducted on the unit cell shown in Figure 1a, consisting of one-wavelength-wide domains including a SiO_2_ substrate (6λ thick), a ZnO piezoelectric film (h_zno_ = 4 μm), an SU-8 adhesive layer (h_SU8_ = 0.5–2 μm), and a SiO_2_ overcoat (6λ thick). A perfectly matched layer (PML) with thickness equal to 2λ was introduced at the bottom of the substrate in order to suppress artificial reflections from the backside of the computational domain. Periodic boundary conditions (PBCs) were imposed on the lateral sides of the unit cell, while the top and bottom boundaries were assumed free and fixed, respectively, as shown in Figure 1b. The eigenfrequency study was performed both on the unit cell shown in Figure 1a and on modified versions of the same structure, including a thin electrically grounded metal plane placed on one side, or on both sides, of the piezoelectric layer.

Four different electroacoustic coupling configurations were investigated depending on the side of the ZnO layer hosting the interdigital transducers (IDTs) and on the presence or absence of a grounded electrical (GE) boundary condition on the opposite side. The four configurations considered were the following: (1) SiO_2_/IDT/ZnO/SU-8/SiO_2_, (2) SiO_2_/ZnO/IDT/SU-8/SiO_2_, (3) SiO_2_/IDT/ZnO/GE/SU-8/SiO_2_, and (4) SiO_2_/GE/ZnO/IDT/SU-8/SiO_2_; the four configurations are hereafter named STF, SFT, STFM, and SMFT, respectively. The electromechanical coupling coefficient, *K*^2^, quantifies the efficiency of electrical-to-acoustic energy conversion. It was evaluated by applying GE boundary conditions to one or both surfaces of the ZnO layer, as illustrated in the insets of Figure 1b. The coupling coefficient was estimated from the phase velocities calculated under electrically free and grounded conditions according to:

$$K^{2}\approx 2\frac{v_{f}-v_{s}}{v_{f}}$$

where $v_{s}=\lambda f_{s}$ and $v_{f}=\lambda f_{f}$ are the phase velocities calculated with and without the thin grounded metal layer, respectively, while $f_{s}$ and $f_{f}$ denote the corresponding eigenfrequencies. The resulting *K*^2^ values provide a quantitative measure of the electroacoustic coupling strength for each investigated configuration.

**Figure 2:**
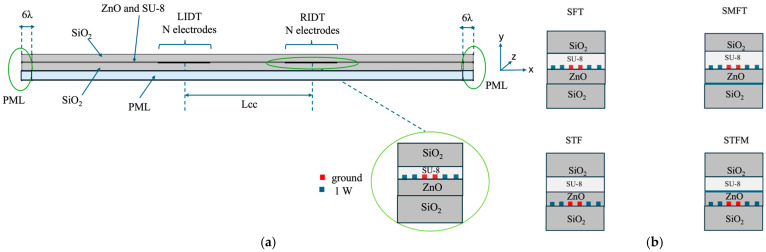
(**a**) The SiO_2_/ZnO/IDT/SU-8/SiO_2_ configuration adopted to study the IAWs propagation; the inset provides an enlarged view of the IDT electrodes and layer stack; (**b**) an enlarged view of the split IDTs (with grounded and terminated electrodes colored in red and blue) in the four coupling configurations.

Frequency-domain simulations were also carried out on a full-scale delay-line structure (shown in Figure 2a) comprising two split-finger IDTs, namely the launching IDT (LIDT) and the receiving IDTs (RIDT), each consisting of N Aluminum (Al) electrodes, 0.15 μm-thick and λ/8 wide. The center-to-center distance between the LIDT and RIDT is defined as $L_{cc}=M\cdot \lambda$, where M is an integer corresponding to the number of acoustic wavelengths between the centers of the two IDTs. The inset in Figure 2a provides an enlarged view of the metal electrodes and highlights the relative positions of the ZnO and SU-8 layers, which are only schematically represented in the main view of the device. It also illustrates the electrical excitation scheme adopted in the simulations, consisting of alternating driven and grounded electrodes. An input power of 1 W was applied to the driven electrodes, while the adjacent electrodes were connected to ground. In the SMFT and STFM configurations, the grounded metal layer located opposite the IDTs does not extend over the entire ZnO surface, but is limited to a region equal in length to the IDTs. This design choice minimizes the direct electrical feedthrough between the launching and receiving transducers while preserving the intended electrical boundary conditions beneath the active transduction region.

The thin-layer approximation was adopted to model the IDTs and grounded planes, thereby significantly reducing the computational burden while preserving simulation accuracy. In all simulations, the material constants of Al, SiO_2_, and ZnO were taken from the Comsol materials library. The SU-8 parameters (mass density: 1190 kg/m^3^, Young’s modulus: E = 4.02 GPa, Poisson’s ratio: ν = 0.22, and relative permittivity: ε_r_ = 3) were taken from Ref. [11]. The mechanical loss factor and dielectric loss tangent were introduced to account for intrinsic material dissipation. Both are dimensionless quantities defined as the ratios between the imaginary and real parts of the corresponding complex material parameters. In COMSOL Multiphysics 6.3, these losses are implemented through imaginary contributions to the elastic and dielectric constitutive parameters, respectively, thereby accounting for intrinsic mechanical and dielectric energy dissipation during acoustic-wave propagation [12,13,14]. ZnO was assigned a dielectric loss tangent of 0.01 and a mechanical loss factor of 0.002, whereas SiO_2_ and SU-8 were assumed to exhibit isotropic mechanical loss factors of 0.001 and 0.01, respectively.

An extremely fine mesh, consisting of physics-controlled triangular elements automatically generated, was employed in all FEM simulations. Low-reflecting boundary conditions and perfectly matched layers (PMLs) were applied at the lateral and bottom boundaries of the computational domain in order to suppress spurious reflections caused by domain truncation. The center-to-center distance between the two IDTs was varied to evaluate the propagation losses (expressed in dB/λ) of the travelling modes. The out-of-plane thickness of the structure, assumed equal to the IDT aperture W, was fixed to 500λ. The simulated transmission characteristics, namely the transmission coefficient S_12_ as a function of frequency, were then analyzed to investigate modal excitation, propagation losses, and the overall delay-line behavior.

### 2.1. Eigenfrequency Study Results

**Figure 3:**
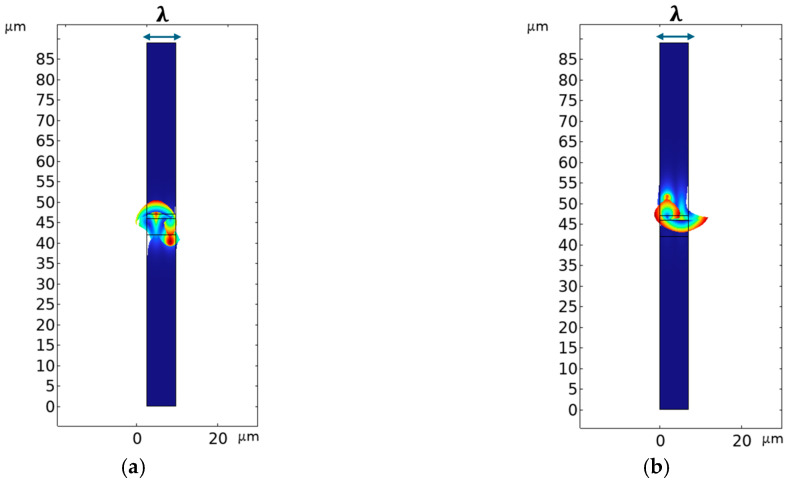

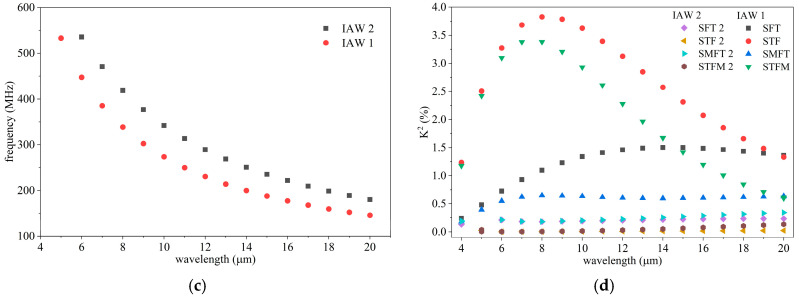
The solid displacement of (**a**) the downward IAW and (**b**) the upward IAW for λ = 8 μm, ZnO and SU-8 layer 4 and 1 μm-thick, respectively; (**c**) the frequency vs. wavelength curves for SU-8 layer 1 μm-thick; (**d**) the *K*^2^ vs. wavelength λ curves of the different coupling configurations, for SU-8 layer 1 μm-thick.

Figure 3a,b shows the solid displacement of the downward and upward IAWs: it is clearly observable that the acoustic field is mainly trapped in the SiO_2_/ZnO substrate for the downward wave, while it is mainly trapped in the ZnO/SU-8/SiO_2_ substrate in the latter case [6]. From the eigenfrequencies *f* of the two modes, the phase velocity of each wave was calculated according to the formula $v_{ph}=f\cdot \lambda$; calculations were performed for different SU-8 layer thicknesses (0.5, 1, and 2 μm), wavelengths λ (from 4 to 20 μm), and for different electrical boundary conditions on the two sides of the ZnO layer (both sides open, both sides shorted, one side shorted and the opposite open). Figure 3c shows, as an example, the IAWs frequency vs. wavelength curves (red and black dots refer to downward and upward IAW, here named as IAW1 and IAW2 for simplicity); Figure 3d shows the *K*^2^ vs. λ curves of the four coupling configurations. All the curves shown in Figure 3c,d were calculated for the SU-8 layer 1 μm-thick.

As observed in Figure 3c,d, the upward-confined IAW (IAW2) propagates faster than the downward-confined IAW (IAW1), since its operating frequency is higher, and the downward IAW exhibits a *K*^2^ significantly larger than that of the upward IAW. This behavior is consistent with the stronger confinement of the electromechanical fields near the SiO_2_/ZnO interface, whereas the upward IAW exhibits a deeper penetration of the acoustic fields into the SiO_2_ overcoat. As a consequence, the propagation losses are strongly influenced by the SU-8-induced modification of the acoustic-field confinement and by the associated leakage mechanisms. It is worth noting that the *K*^2^ values of both IAWs can exceed those of the SAWs propagating in the reference SiO_2_/ZnO (4 µm)/IDT/air structure, which exhibits *K*^2^ values of approximately 1.12% and 3.7% for the Rayleigh and Sezawa modes at ZnO thickness-to-wavelength ratios of 0.4 and 0.375, respectively [7]. Table 1 summarizes the maximum *K*^2^ values obtainable with the two waves, and the corresponding wavelength and SU-8 thickness.

### 2.2. Frequency Domain Study

The S_12_ versus frequency responses of the IAW-based delay lines were calculated at the wavelength and SU-8 thickness values corresponding to the maximum *K*^2^ of each coupling configuration, as summarized in Table 1. These simulations were performed: (i) for different values of *N*, in order to identify the design conditions leading to reduced insertion losses; and (ii) for different values of *L_cc_*, in order to evaluate the propagation loss α of the IAWs.

**Figure 4:**
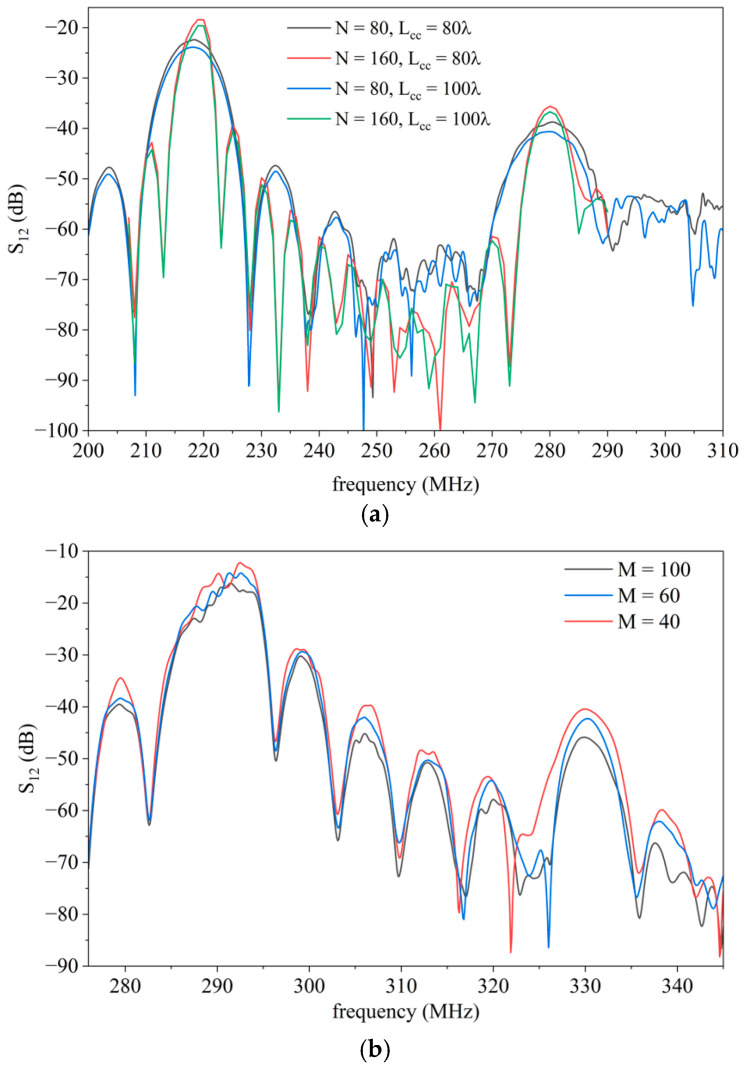

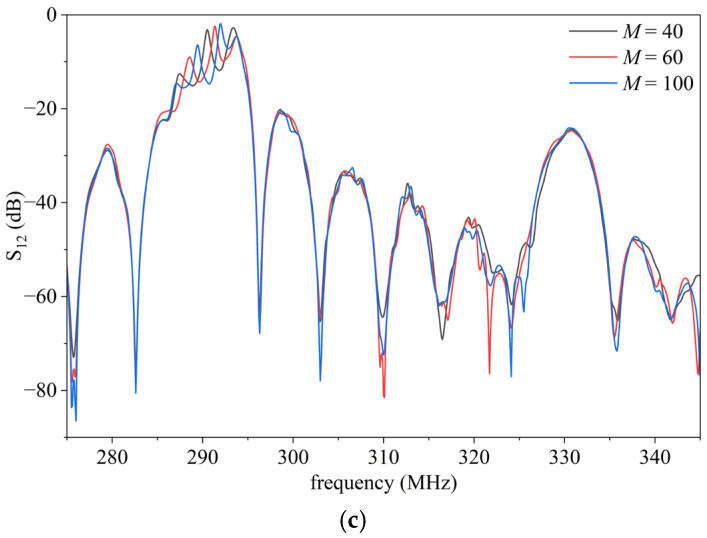
The S_12_ vs. frequency curves of the IAWs travelling in (**a**) the SFT configuration with SU-8 = 0.5 μm, λ = 13 μm, *N* = 80 and 160, W = 500λ, and L_cc_ = 80λ and 100λ; (**b**) the STF configuration with λ = 9 μm, SU-8 2 μm, for *M* ranging from 40 to 100 wavelengths; (**c**) the same curves as in (**b**) but obtained with all material loss parameters set to zero.

Figure 4a shows, as an example, the S_12_ versus frequency response of the SFT delay lines, where the two peaks corresponding to the two IAWs are clearly distinguishable. The reported curves refer to devices with λ = 13 μm and an IDT aperture of W = 500λ, for different numbers of IDT electrodes (*N* = 80 and 160) and different distances between the two IDTs (*M* = 80 and 100 wavelengths). By comparing the curves of Figure 4a, it appears evident that an increase in the number *N* of IDT electrodes leads to reduced insertion losses and narrower bandwidths, owing to the enhanced electromechanical transduction efficiency and increased frequency selectivity of the transducers. At the same time, the oscillation peaks become more pronounced and spectrally better resolved, thereby improving the discrimination between the upward and downward IAW modes. The wave propagation loss α (expressed in dB/λ) listed in Table 1 was extracted as the slope of the S_12_ value at the synchronous frequency of each mode versus the number of wavelengths separating the two IDTs. The estimated propagation loss values may be affected by some uncertainties arising from the presence of some ripples in the S_12_ vs. frequency curves. Figure 4b shows, as an example, the S_12_ vs. frequency curve for STF configuration with λ = 9 μm, SU-8 2 μm, *N* = 160, for *M* ranging from 40 to 100 wavelengths. We can notice that the insertion loss (IL) of the STF configuration is lower than that of the SFT configuration, in accordance with the larger *K*^2^.

To assess the contribution of the adopted material loss parameters to the delay-line insertion loss, an additional simulation was performed with all material losses set to zero, while keeping the device geometry, boundary conditions, and PML configuration unchanged. The lossless S_12_ responses calculated for different values of *M* are shown in Figure 4c and should be compared with the corresponding responses of the same device configurations, including material losses, shown in Figure 4b. The difference between the lossy and lossless simulations provides an estimate of the contribution of intrinsic material damping. In the lossless case, the insertion loss observed in the S_12_ curves shows only a weak dependence on the IDT-to-IDT separation, indicating that propagation-related leakage is limited over the investigated distances; the residual insertion loss observed can be attributed to acoustic leakage and radiation, incomplete electromechanical transduction, impedance mismatch, and reflections between the transmitting and receiving IDTs, which cause the ripples observed on the S_12_ transmission peaks. As shown in Figure 4c, the lossless delay lines exhibit IL of about 2–4 dB, while when the material losses are included (Figure 4b), IL increases by approximately 10 dB. Therefore, the comparison between the lossy and lossless simulations indicates that intrinsic material dissipation provides the dominant contribution to the simulated insertion loss, whereas leakage and radiation mechanisms make a smaller contribution. The adopted material loss parameters represent a conservative assumption, intended to avoid overestimating the device performance.

Figure 4 confirms that the IDT design strongly influences the overall device performance: in particular, increasing the number *N* of IDT electrodes improves the electromechanical conversion efficiency and reduces the acoustic power radiated outside the main resonance band, although at the expense of a narrower operational bandwidth. Moreover, the two S_12_ peaks exhibit different amplitudes, consistent with the different *K*^2^ values predicted by the eigenfrequency analysis, with the downward IAW showing the highest excitation efficiency. Despite the different insertion losses associated with the two modes, the obtained responses demonstrate that both IAWs can be effectively excited and detected by conventional IDT configurations, thereby confirming the feasibility of IAW-based delay lines.

**Figure 5:**
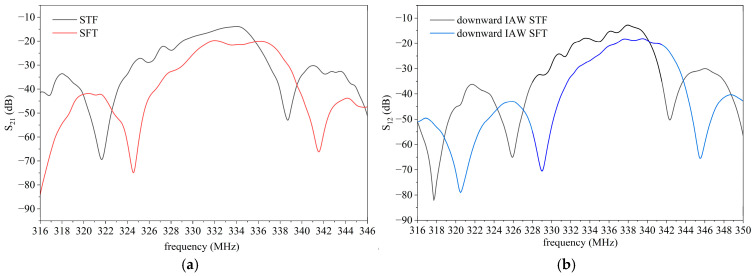
The S_12_ vs. frequency curves of the (**a**) Rayleigh wave and (**b**) downward IAW travelling in the SFT and STF coupling configurations, with ZnO 4 μm-thick, λ = 8 μm (same split finger IDT geometry), *N* = 160, *M* = 60 and W = 500λ.

Finally, the frequency responses of the IAW devices were compared with those of the Rayleigh wave propagating along the surface of the reference SiO_2_/ZnO (4 µm)/air structure in order to assess the potential of IAW-based configurations as an alternative to conventional SAW technology, particularly for applications requiring reduced sensitivity to environmental perturbations and potentially enabling packageless operation. Figure 5 shows the S_12_ frequency responses of the SAW- and IAW-based delay lines with λ = 8 μm, split-finger IDTs, a 4 μm-thick ZnO layer, SU-8 layer 1 μm-thick, *N* = 160, *M* = 60, and W = 500λ. The same material-loss parameters were used in both the SAW and IAW simulations.

The plots in Figure 5 highlight that the IAWs operate at higher frequencies than the corresponding SAWs, indicating larger phase velocities of the interface-confined modes. At the same time, the insertion losses remain comparable for both wave types and coupling configurations, demonstrating that IAW-based devices can achieve RF performance similar to that of conventional SAW delay lines despite their different field-confinement mechanisms.

## 3. Discussion

In the available literature, only a limited number of studies have addressed IAW configurations based on piezoelectric thin films. T. Irino and collaborators [3] reported theoretical and experimental IAW delay lines based on SiO_2_/ZnO/SiO_2_ structures. The reported devices exhibited good performance, with *K*^2^ = 1.8%, a propagation loss α = 0.081 dB/λ for L_cc_ = 100λ and λ = 10 μm, an aperture of 1 mm, and an insertion loss of approximately 17 dB. However, the fabrication process required the IDTs to be buried beneath a 3.8 μm-thick ZnO layer and covered by a thick SiO_2_ overcoat approximately 23 μm-thick, whose deposition involved relatively long and technologically demanding fabrication processes lasting many hours.

In references [15,16,17,18], the SAW propagation in c-ZnO/Si, 128° YX-LiNbO_3_, and ZnO/128° YX-LiNbO_3_ substrates covered by thick protective AlN or Al_2_O_3_ overlayers was experimentally investigated. These protective layers, often several acoustic wavelengths thick, were introduced to isolate the devices from environmental perturbations and enable packageless operation.

Reference [19] investigated boundary-wave propagation in PZT/adhesive/PZT and PZT/adhesive/Corning-glass structures by using an α-cyanoacrylate instant adhesive as the bonding layer. The authors reported that the differences observed between the theoretical and experimental results were mainly associated with the non-uniform characteristics of the adhesive layer.

In Ref. [6], IAW propagation in SiO_2_/ZnO/SU-8/SiO_2_ multilayer structures with a 4 μm-thick ZnO layer and a 6 μm-thick SU-8 layer was experimentally investigated for λ = 80 μm, *N* = 80 (split IDTs) and *M* = 60. In that work, both the downward- and upward-confined IAWs were experimentally observable, although the corresponding insertion losses were as large as approximately 80 dB.

The present study significantly improves upon the results reported in Ref. [6] and demonstrates that reducing the acoustic wavelength from several tens of micrometers down to only a few micrometers, together with decreasing the SU-8 thickness from approximately 6 μm down to 0.5–2 μm, leads to a substantial enhancement of the electromechanical coupling coefficient of the IAWs: in particular, the *K*^2^ values increase from approximately 0.11% and 0.035% up to nearly 4.0% and 1.2% for the downward- and upward-confined IAWs, respectively.

In contrast with Ref. [3], the present work demonstrates comparable acoustic performance using a considerably simpler low-temperature bonding approach, where a thin SU-8 adhesive layer is employed to attach a bulk fused-silica overcoat onto the SiO_2_/ZnO/IDT substrate. The fabrication process is technologically straightforward, since SU-8 can be easily spin-coated onto the fused-silica overcoat and subsequently bonded under mild pressure, followed by a soft bake at 95 °C.

The downward-confined IAW systematically exhibits a significantly larger *K*^2^ than the upward-confined IAW. This behavior is physically consistent with the stronger confinement of the electromechanical field near the upper region of the SiO_2_/ZnO substrate, where the interaction with the piezoelectric ZnO layer is more efficient. In particular, the downward IAW is mainly localized within the SiO_2_/ZnO region and exhibits only a small evanescent tail extending into the SU-8/SiO_2_ overcoat, whereas the upward IAW is predominantly confined within the ZnO/SU-8/SiO_2_ region, with only a weak evanescent tail penetrating into the SiO_2_ substrate. As a consequence, the electromechanical interaction associated with the upward IAW becomes weaker, leading to reduced excitation efficiency, lower *K*^2^ values, and larger insertion losses in the calculated scattering-parameter responses.

The frequency-domain simulations further demonstrate that the same split-finger IDT geometry is capable of simultaneously exciting and detecting both the downward- and upward-confined IAWs, despite their significantly different electromechanical coupling coefficients. Since the upward-confined IAW exhibits a lower *K*^2^ than the downward-confined IAW, its optimal excitation would likely require a transducer geometry specifically optimized for the weaker-coupled mode. More generally, the insertion losses of both IAWs could be further reduced through dedicated optimization of the IDT geometry and electrical matching conditions. In particular, increasing the number of IDT finger pairs and optimizing the acoustic aperture would improve the electromechanical conversion efficiency, especially for the upward-confined IAW. In addition, improved impedance matching and the use of thinner or mechanically stiffer bonding layers, such as spin-on-glass, could further reduce both transduction and propagation losses.

The present study indicates that SAW- and IAW-based delay lines operate in comparable frequency ranges, while the IAWs propagate with higher phase velocities and exhibit insertion and propagation losses of the same order of magnitude as those of conventional SAWs. However, since insertion loss is also strongly affected by the IDT geometry, impedance matching, and excitation efficiency, the reported values should not be regarded as the ultimate performance limits of the proposed structures. Further reductions in the insertion loss of the IAWs are therefore expected through several strategies, such as:

(i) optimized transducer layouts;
(ii) dedicated impedance-matching strategies;
(iii) the use of less lossy adhesive layers;
(iv) the adoption of more efficient piezoelectric materials.

The operating principle and architecture of the IAW-based device were previously validated experimentally in Ref. [6]. In the present work, the geometrical and electrical design parameters are systematically varied and optimized. Since the original device was successfully demonstrated under non-optimized conditions, the experimentally validated structure reported in Ref. [6] provides a physically grounded reference platform for investigating configurations expected to offer improved performance.

The aim of the present manuscript is therefore to determine how the geometrical and electrical parameters of the IAW delay line affect its electromechanical coupling, acoustic-field confinement, modal response, and transmission characteristics, and to identify design conditions that may improve its overall performance. As the predicted improvements have not yet been experimentally validated, the results should be regarded as design guidelines for the future fabrication and characterization of optimized bonded IAW devices rather than as experimentally demonstrated performance gains. Nevertheless, the study provides a systematic framework for identifying promising multilayer geometries and electrical coupling configurations for future sensing and telecommunications applications.

## 4. Conclusions

This work investigated the propagation of interface acoustic waves in bonded SiO_2_/ZnO/SU-8/SiO_2_ multilayer structures through combined eigenfrequency and frequency-domain analyses. The results demonstrated the coexistence of two distinct interface acoustic modes propagating within the same structure, namely an upward-confined and a downward-confined IAW. Both modes originate from the evolution of the Rayleigh wave supported by the reference SiO_2_/ZnO structure following the introduction of the SU-8/SiO_2_ overcoat. The analyses showed that the propagation characteristics and electromechanical coupling of the IAWs strongly depend on the multilayer geometry, particularly on the thicknesses of the ZnO and SU-8 layers. By scaling the acoustic wavelength down to a few micrometers and optimizing the multilayer configuration, substantial improvements in electromechanical coupling and insertion loss were numerically predicted compared with previously investigated large-wavelength structures. The results also demonstrated that conventional split-finger IDTs can efficiently excite and detect both interface-wave modes, and that the proposed multilayer structures can exhibit simulated performance comparable to that of conventional SAW devices while relying on a comparatively simple fabrication approach based on SU-8 bonding. In contrast to previously reported IAW technologies requiring thick sputtered overcoats or additional protective layers, the proposed structures employ a low-temperature and potentially lower-cost bonding process compatible with compact device fabrication. Therefore, the present study indicates that bonded SiO_2_/ZnO/SU-8/SiO_2_ multilayer structures represent a promising platform for future acoustic-wave devices, particularly for sensing and telecommunications applications requiring compactness, robustness, and reduced packaging complexity. Because the acoustic energy is guided along buried interfaces, these structures may provide an effective route toward compact and potentially packageless acoustic-wave devices that are less susceptible to environmental perturbations than conventional surface-confined SAW devices. The proposed device designs have not yet been experimentally validated and should therefore be regarded as design guidelines for the future fabrication and characterization of optimized bonded IAW devices. Nevertheless, the results provide a systematic framework for identifying promising multilayer geometries and coupling configurations. Preliminary studies of IAW resonators have yielded promising results, and further work is currently in progress to optimize their design and experimentally assess their performance. Additional theoretical and experimental investigations will also be required to clarify the physical mechanisms governing IAW emergence, confinement, and propagation loss, as well as to evaluate alternative piezoelectric and adhesive materials and optimized electrical boundary conditions for practical device implementation.

## Figures and Tables

**Table 1:** Maximum *K*^2^ obtainable with the two waves (IAW1 and IAW2 denote the downward and upward IAWs, respectively), the wavelength, the SU-8 thickness and the propagation loss.

Coupling Config./IAW	SU-8 Thickness (μm)	λ (μm)	*K*^2^ (%)	α (dB/λ)
STF/IAW1	2.0	9	3.70	0.057
STF/IAW1	0.5	8	3.63	0.052
STF/IAW1	1.0	8	3.90	0.0547
STFM/IAW1	0.5	7	3.24	0.070
STFM/IAW1	1.0	8	3.40	0.080
SFT/IAW1 and IAW2	0.5	13	1.40	0.070 and 0.063

## Data Availability

The original contributions presented in this study are included in the article. Further inquiries can be directed to the corresponding author.

## Abbreviations

The following abbreviations are used in this manuscript:

IAW	Interface acoustic wave
SAW	Surface acoustic wave
IDT	Interdigital transducer
